# Clinical and Behavioral Outcomes During 4 Weeks of Home-Based Self-Administered Transcranial Direct Current Stimulation in Perinatal Women With Depressive Symptoms: Open-Label Exploratory Pilot Study

**DOI:** 10.2196/56454

**Published:** 2026-03-12

**Authors:** Sehwan Park, Gangho Do, Jaesub Park, Min-Kyoung Kim, Sra Jung, Hyun-Ju Kim, Hee Young Cho

**Affiliations:** 1 Medical Research Team Digital Medic Co, Ltd Seoul Republic of Korea; 2 Digital Medic Co, Ltd Seoul Republic of Korea; 3 Department of Psychiatry Yongin Severance Hospital Yonsei University College of Medicine Yongin, Seoul Republic of Korea; 4 Department of Psychiatry CHA Ilsan Medical Center CHA University Goyang-si, Gyeonggi-do Republic of Korea; 5 Department of Psychiatry CHA Bundang Medical Center CHA University Seongnam, Gyeonggi-do Republic of Korea; 6 Department of Obstetrics and Gynecology Seoul National University College of Medicine Seoul National University Hospital Seoul Republic of Korea; 7 Institute of Reproductive Medicine and Population Medical Research Center Seoul National University Seoul Republic of Korea

**Keywords:** transcranial direct current stimulation, tDCS, perinatal depression, wearable devices, digital phenotyping, behavioral activation, pilot study

## Abstract

**Background:**

Depression during the perinatal period poses significant risks to both maternal and infant health. Although transcranial direct current stimulation (tDCS) has shown promise as a safe and well-tolerated intervention for perinatal depression, empirical evidence remains limited, and no prior study has integrated clinical outcomes with continuous objective behavioral monitoring.

**Objective:**

This pilot study aimed to evaluate the feasibility and preliminary effects of home-based tDCS combined with wearable monitoring in women with perinatal depression and to explore the correlation between behavioral and clinical measures.

**Methods:**

In total, 38 perinatal women completed a 4-week self-administered tDCS protocol targeting the left dorsolateral prefrontal cortex (DLPFC) with 30-minute daily sessions. Participants continuously wore a wrist device (Fitbit Inspire 2) that passively collected data on the step count, distance traveled, calorie expenditure, and heart rate. Depressive symptoms were assessed at baseline (week 0), midintervention (week 2), and postintervention (week 4) using the Montgomery-Åsberg Depression Rating Scale (MADRS) and the Beck Depression Inventory (BDI). Linear mixed models (LMMs) and nonparametric tests were used to evaluate changes over time, and correlations between behavioral and clinical variables were analyzed.

**Results:**

Significant reductions were observed in depressive symptoms from baseline to postintervention. Mean MADRS scores decreased from 21.3 (SD 5.2) at baseline to 12.3 (SD 8.5) at week 4 (P<.001), and mean BDI scores decreased from 24.8 (SD 11.4) at baseline to 16.1 (SD 11.2) at week 4 (P<.001). Physical activity markers increased significantly: from baseline to week 4, the mean daily step count increased from 2366.8 (SD 2293.0) to 6278.7 (SD 5026.8) steps (P<.001), the mean distance traveled daily increased from 1564.6 (SD 1528.4) to 4105.9 (SD 3117.6) m (P<.001), and the mean daily calorie expenditure increased from 1130.6 (SD 759.9) to 1834.9 (SD 458.1) kcal (P<.001). However, the mean resting heart rate decreased from 85.9 (SD 9.0) to 80.4 (SD 7.0) bpm (P<.01). Exploratory correlation analyses revealed that BDI score reductions, but not MADRS changes, correlated significantly with increases in physical activity indicators (step count: r=0.401, P=.02 at week 2; r=0.351, P=.04 at week 4).

**Conclusions:**

Results showed that 4 weeks of home-based, self-administered tDCS are feasible, well tolerated, and associated with improvements in depressive symptoms and objective behavioral activity among perinatal women. Self-reported symptom changes show stronger correlations with behavioral outcomes compared to clinician-rated scores. Although the lack of a control group precludes causal inference, these findings support the feasibility of integrating wearable monitoring with tDCS protocols and warrant further validation in randomized sham-controlled trials.

## Introduction

Perinatal depression, encompassing both antenatal and postpartum periods, is among the most prevalent mental health conditions affecting women during childbearing years [1]. Studies estimate that 15%-20% of women experience clinically significant depressive symptoms during this period, often extending for months if left untreated [2]. These mental health difficulties not only affect maternal emotional well-being but are also associated with increased risk for obstetric complications, such as preeclampsia, preterm birth, and low birth weight, as well as disruptions in maternal-infant bonding and adverse long-term outcomes in child development [3-6].

Despite the high prevalence and clinical relevance of perinatal depression, access to appropriate care remains limited [7]. Pregnant and postpartum women often face significant barriers to seeking mental health treatment, including stigma, limited service availability, and safety concerns about pharmacotherapy [7,8]. Psychological interventions, such as cognitive behavioral therapy and interpersonal therapy, are effective but require intensive time commitments and access to trained professionals. These requirements may be difficult to meet during pregnancy or early motherhood, when competing demands are substantial [8,9]. These challenges have driven increasing interest in developing nonpharmacological and more accessible interventions, particularly those that can be delivered safely at home during the perinatal period [10].

Transcranial direct current stimulation (tDCS) is a noninvasive brain stimulation method that applies weak electrical currents through scalp electrodes to modulate cortical excitability and induce neuroplastic changes [11]. A substantial body of evidence across psychiatric and neurological populations indicates that tDCS is generally well tolerated, with adverse event (AE) and dropout rates comparable to sham stimulation [12,13]. Importantly, early clinical investigations suggest that tDCS may also be feasible and safe in perinatal populations. In one pilot randomized controlled trial [14], symptom reductions were observed in pregnant women with major depressive disorder (MDD), with no major maternal or neonatal complications. Another open-label study during pregnancy [15] demonstrated similar improvements, and a case report [16] described successful tDCS monotherapy in a perinatal patient. A systematic review [17] further concluded that noninvasive neurostimulation appears promising and well tolerated during pregnancy. Collectively, these studies indicate that tDCS represents a feasible, safe, and potentially effective treatment option for perinatal depression.

Most prior studies have focused on symptom reduction using validated clinician-rated or self-reported measures, such as the Montgomery-Åsberg Depression Rating Scale (MADRS), the Hamilton Depression Rating Scale (HAMD), or the Beck Depression Inventory (BDI) [18,19]. Although these tools are essential for evaluating depression severity, they may offer limited insight into functional changes in everyday behavior [19,20]. Real-world functioning, including physical activity, sleep, and physiological regulation, is increasingly recognized as an important dimension of treatment response, particularly in perinatal populations where the ability to maintain activity and caregiving responsibilities is directly linked to maternal and infant health [21,22].

Wearable devices and digital phenotyping methods, involving moment-by-moment quantification of individual-level behavioral patterns using data from personal devices, now provide opportunities to objectively measure such outcomes [23]. Recent studies on depression have demonstrated that wearable-derived metrics, such as the step count, distance traveled, and heart rate, are associated with self-reported mood and stress, offering ecologically valid markers of behavioral and physiological change [23-26]. However, to the best of our knowledge, no prior study has simultaneously evaluated associations between tDCS and both clinical symptom scales and wearable-based behavioral indicators in perinatal women [27].

In summary, although emerging evidence supports the feasibility and safety of tDCS for depressive symptoms during pregnancy, little is known about its integration with real-world behavioral monitoring or its applicability in home-based settings. To address these gaps, we conducted an open-label exploratory pilot study designed to primarily evaluate feasibility and exploratory associations, combining 4 weeks of daily tDCS with continuous wearable monitoring and standardized clinical assessments in perinatal women. This study primarily evaluated the feasibility and preliminary effects of home-based, self-administered tDCS on depressive symptoms and wearable-derived behavioral outcomes and further explored the association of clinician-rated (MADRS) and self-reported (BDI) symptom measures with behavioral indicators. Ultimately, the findings may inform the development of integrated digital neuromodulation platforms for perinatal depression.

## Methods

### Trial Design

This study was a 4-week, open-label, multicenter pilot trial conducted across four medical centers in South Korea to assess the feasibility, safety, and preliminary associations between home-based self-administered tDCS and clinical and behavioral outcomes in perinatal women with depressive symptoms. Trial registration was not applicable as this was an exploratory pilot study. Participants were recruited on a rolling basis without a formal sample size calculation, as the study was designed to evaluate feasibility and inform power estimates for future trials.

### Participants

In total, 38 participants were enrolled during the pilot phase, of whom 36 (95%) completed follow-up to week 4 and provided analyzable data. Participants were recruited through internet-based advertisements and onsite recruitment at four medical centers in South Korea: the Seoul National University Hospital (Seoul), the Yongin Severance Hospital (Yongin), the CHA University Bundang Medical Center (Seongnam), and the Ilsan CHA Hospital (Goyang-si). Recruitment occurred from November 2023 to March 2025 through outpatient clinics in obstetrics and psychiatry.

The target population included perinatal women aged 19-49 years, encompassing those with infertility, planning pregnancy, currently pregnant, or in the postpartum period. Inclusion criteria were as follows:

- Mild-to-moderate MDD, defined as meeting the diagnostic criteria for unipolar, nonpsychotic MDD according to the *Diagnostic and Statistical Manual of Mental Disorders, Fifth Edition* (DSM-5), with severity indicated by a BDI-II score of 18-28 or a MADRS score of 14-34
- Current MDD episode confirmed by the Mini International Neuropsychiatric Interview (MINI)
- Sufficient proficiency in Korean to understand study procedures and complete questionnaires
- Ability to provide informed consent
- Access to a smartphone compatible with the study app
- Willingness and ability to comply with the 4-week intervention protocol

Exclusion criteria were as follows:

- History of psychiatric comorbidities, including post-traumatic stress disorder or obsessive-compulsive disorder
- Diagnosis of bipolar or psychotic MDD
- History of seizure disorder or neurological conditions associated with increased risk for seizures that would make participation unsafe, as judged by the investigator
- MADRS item 10 (suicidal thoughts) score≥5 or suicide attempt within 6 months prior to screening
- Imminent suicide risk requiring psychiatric hospitalization, as judged by the investigator
- Scalp abnormalities, inflammatory reactions, or dermatological conditions preventing proper electrode placement
- Contraindications to noninvasive brain stimulation (eg, metal implants in the head, cardiac pacemakers, implantable cardioverter-defibrillators, or other implanted medical devices)
- Clinically significant cardiovascular, gastrointestinal, respiratory, endocrine, or central nervous system disorders interfering with device application or efficacy assessment
- Participation in another clinical trial within 30 days prior to screening
- Receipt of tDCS within 6 months prior to screening
- Any other clinically significant finding deemed inappropriate for study participation by the investigator

At visit 1 (V1; baseline, week 0), eligible participants completed demographic and clinical assessments. Follow-up evaluations were conducted at visit 2 (V2; week 2) and visit 3 (V3; week 4).

### Ethical Considerations

This study was approved by the Institutional Review Boards (IRBs) of the Seoul National University Hospital (IRB #2307-135-1452); the Yongin Severance Hospital (IRB #2023-07-004); the CHA Bundang Medical Center, CHA University College of Medicine (IRB #2023-08-024); and the Ilsan CHA Hospital (IRB #2023-07-004). All participants provided written informed consent before enrollment. Participants were fully informed about the study’s purpose, procedures, and potential risks, as well as their right to withdraw at any time without consequence. All study procedures complied with the principles of the Declaration of Helsinki and local ethical guidelines. To ensure privacy and confidentiality, all data were deidentified before analysis and securely stored on encrypted servers, accessible only to authorized research staff. Participants received a financial compensation of Korean Republic Won (KRW) 20,000 (~US $14) per visit (V1, V2, and V3).

### Transcranial Direct Current Stimulation Intervention

Participants self-administered active tDCS once per calendar day for 4 weeks in an open-label design. There were no restrictions on the time of day, but multiple sessions within the same day were not permitted. Stimulation was delivered using a Conformité Européenne (CE)-certified portable stimulator (YMS-201B+/201BS+, MINDD STIM+; Ybrain Inc), which is classified as a class III medical device (psychotherapy brain electrical stimulator) approved by the Ministry of Food and Drug Safety (MFDS, Korea; product classification code A16180.02). The anode was positioned over the left dorsolateral prefrontal cortex (DLPFC; F3 according to the 10-20 electroencephalogram [EEG] system) and the cathode over the right DLPFC (F4), corresponding to Brodmann areas 9 and 46.

Each stimulation session lasted 30 minutes, consisting of a 30-second ramp-up phase, 29 minutes of active stimulation, and a 30-second ramp-down phase. Current intensity was set to either 2 or 1.5 mA, depending on participant tolerability. If participants demonstrated sensitivity to the 2 mA current during the initial sessions, the stimulation intensity was reduced to a minimum of 1.5 mA, and such adjustments were documented in the case report form.

Participants were instructed to complete one session per day, with a minimum of five and up to seven sessions per week. Thus, over the 4-week intervention period, participants could complete between 20 and 28 sessions. Saline-soaked sponge electrodes were used to ensure stable conductivity and to minimize skin irritation. No concomitant interventions were introduced during the study, and concomitant medications were permitted if clinically indicated and recorded in the case report form.

All participants attended an initial training session with study staff to ensure correct electrode placement, proper device handling, and safe operation. Written instructions and pictorial guides were provided for home use. Participants were instructed to synchronize the stimulation device with a dedicated smartphone app, which recorded adherence and sent automated reminders. Device logs and transmitted data ensured objective monitoring of compliance throughout the study.

Safety was monitored throughout the intervention period. Study staff asked participants about any adverse sensations or symptoms after each session, and investigators reviewed these reports weekly to identify potential AEs. All events were documented in the case report form and evaluated for seriousness and relatedness to the stimulation.

### Behavioral Assessments

Behavioral outcomes were continuously assessed using a wrist-wearable device (Fitbit Inspire 2, Fitbit Inc). Four indicators were monitored: daily step count, distance traveled, calorie expenditure, and average heart rate. All wearable-derived variables were obtained from data collected via the Fitbit application programming interface (API) and aggregated by the investigators into daily mean values for analysis.

The step count, distance traveled, and calorie expenditure were derived from the Fitbit activity intraday endpoints, which provide minute-level measures of physical activity recorded while the device is worn. Distance traveled estimates were calculated by the device based on step counts and user-specific stride length parameters. Calorie expenditure reflects the total energy expenditure, including periods of physical activity as well as resting metabolic processes, as defined by the manufacturer.

Heart rate data were obtained from the Fitbit activities-heart-intraday endpoint, which provides minute-level heart rate values derived from photoplethysmography-based measurements. These intraday heart rate values represent physiological measurements collected while the device is worn and were summarized as the daily mean heart rate for each participant.

Baseline behavioral data were collected from the screening visit for the baseline assessment (1-3 days), whereas week 2 and week 4 data represented approximately 2-week collection periods preceding each assessment. Devices were worn continuously throughout the 4-week intervention period, and data were automatically uploaded to the study database through the Fitbit API. These measures provided objective and ecologically valid indices of participants’ daily physical activity and physiological state [28]. Detailed definitions of wearable-derived features, corresponding Fitbit API endpoints, temporal resolution, and aggregation procedures are provided in Multimedia Appendix 1.

### Clinical Assessments

Clinical outcomes were evaluated at baseline (week 0), midintervention (week 2), and postintervention (week 4). Depressive symptoms were assessed using two validated instruments. MADRS is a clinician-administered measure consisting of 10 items rated on a scale from 0 to 6 (total range 0-60), with higher scores indicating more severe depressive symptoms [29,30]. The Korean version of the Beck Depression Inventory-II (K-BDI-II) is a 21-item self-report questionnaire scored on a scale of 0 to 3 per item (total range 0-63), with higher scores reflecting greater depression severity [31,32]. Both scales have been validated for use in clinical and research settings and are sensitive to changes in depressive symptomatology over short time intervals.

Demographic and clinical background information, including obstetric status, psychiatric history, medical history, and medication use, were collected at baseline through structured questionnaires. Perinatal status was recorded in detail, including pregnancy trimester or postpartum duration, to allow stratification of participants according to the reproductive stage.

### Data Analysis

All statistical analyses were performed using Python 3 (Python Software Foundation) with the *pandas*, *stats models*, and *scipy* packages. Descriptive statistics were used to summarize baseline sociodemographic and clinical characteristics. Continuous variables were presented as means (SDs) and categorical variables as frequencies and percentages. Missing data were handled using available-case analysis. For heart rate, missing values occurred primarily due to device removal or loose fit during sleep, which we considered to represent missing at random with respect to treatment response. Heart rate data were assumed to be missing at random for analytical purposes; however, no formal statistical test was conducted to verify this assumption. The number of valid observations is reported for each analysis.

For clinical outcomes, changes across time points (weeks 0, 2, and 4) were analyzed using linear mixed models (LMMs), with participant-level random intercepts and time as a fixed effect [33]. Results are reported as regression coefficients (β), SEs, 95% CIs, and *P* values.

For behavioral outcomes (step count, distance traveled, calorie expenditure, and heart rate), both LMMs and nonparametric tests were applied. Friedman tests were conducted to examine overall time effects, followed by pairwise Wilcoxon signed-rank tests. For these post hoc comparisons, *P* values were adjusted using Bonferroni correction to control for multiple testing. Effect sizes were calculated as Cohen d for descriptive purposes. It should be noted that effect sizes in within-subject pre-post designs may overestimate treatment-related effects in the absence of a control group and, thus, should be interpreted cautiously.

LMMs were applied to examine changes over time, with time as a fixed effect and participant as a random effect. Covariates were not included in the primary models; age-adjusted models were conducted as sensitivity analyses and are presented in Multimedia Appendix 2.

Associations between changes in clinical outcomes and behavioral indicators were assessed using Spearman rank correlation coefficients (r) with corresponding *P* values. Change scores were calculated for three intervals: week 2 vs week 0, week 4 vs week 0, and week 4 vs week 2.

To further account for multiple testing, false discovery rate (FDR) correction was explored for overall nonparametric analyses (Friedman tests); however, the main results are presented with Bonferroni-adjusted values for pairwise comparisons. All statistical results, including test statistics, *P* values, effect sizes, and 95% CIs, are reported.

## Results

### Participant Enrollment

A total of 38 perinatal women were enrolled in the study, of which 2 (5%) were excluded from the analyses: 1 (50%) participant did not provide any behavioral data, and 1 (50%) participant had no data available after week 2. Accordingly, the data of 36 (95%) participants were included in subsequent analyses.

### Baseline Characteristics

Baseline demographic and clinical characteristics were assessed for all 38 (100%) participants. The mean age was 34.8 (SD 4.3) years, and the mean height was 163.2 (SD 6.2) cm. The majority of participants were married (35/38, 92%). In terms of occupation, 14 (37%) participants were homemakers, 10 (26%) were professionals, and 8 (21%) were white-collar workers, with smaller proportions engaged in services (2/38, 5%), self-employment (2/38, 5%), technical work (1/38, 3%), or as students (1/38, 3%).

The mean systolic and diastolic blood pressures were 116.1 (SD 13.4) and 70.0 (SD 11.5) mmHg, respectively. The mean pre-pregnancy weight and BMI were 60.9 (SD 10.1) kg and 22.9 (SD 4.2), whereas the current weight and BMI at baseline were 66.0 (SD 13.4) kg and 24.8 (SD 5.1). All 38 (100%) participants were nonsmokers, and 2 (5%) reported current alcohol use.

Regarding perinatal status, 11 (29%) participants were postpartum, 20 (53%) were pregnant (n=9, 45%, in the first trimester, n=8, 40%, in the second trimester, and n=3, 15%, in the third trimester), and 7 (18%) either had infertility or were planning pregnancy. The mean gestational age at participation among pregnant participants was 16 weeks and 6 days (SD 70.7 days). The postpartum period was defined as within 12 months after delivery and was, on average, 47.2 (SD 36.0) days after delivery.

These baseline characteristics are summarized in Tables 1 and 2.

**Table 1:** Baseline demographic characteristics of study participants (N=38).

Characteristics		Value
Age, years, mean (SD)		34.8 (4.3)
**Occupation, n (%)**		
	Homemakers	14 (37)
	White-collar workers	8 (21)
	Services	2 (5)
	Professionals	10 (26)
	Technical jobs	1 (3)
	Students	1 (3)
	Self-employed	2 (5)
**Marital status, n (%)**		
	Married	35 (92)
	Divorced	1 (3)
	Single mother	2 (5)
Number of offspring, mean (SD)		1.6 (0.8)^a^

^a^Data of 37 (97%) participants.

**Table 2:** Baseline clinical characteristics of study participants (N=38).

Characteristics			Participants, n (%)		Mean (SD)
**Physical characteristics**					
	Systolic blood pressure (mmHg)	37 (97)		116.1 (13.4)	
	Diastolic blood pressure (mmHg)	37 (97)		70.0 (11.5)	
	Pre-pregnancy weight (kg)	32 (84)		60.9 (10.1)	
	Weight (kg)	37 (97)		66.0 (13.4)	
	Height (cm)	38 (100)		163.2 (6.2)	
	Pre-pregnancy BMI	32 (84)		22.9 (4.2)	
	Current BMI	33 (87)		24.8 (5.1)	
**Smoking**					
	Yes	0		—^a^	
	No	38 (100)		—	
**Alcohol use**					
	Yes	2 (5)		—	
	No	36 (95)		—	
**Gestational status**					
	Planning pregnancy	2 (5)		—	
	First trimester (weeks 0-13)	9 (24)		—	
	Second trimester (weeks 14-28)	8 (21)		—	
	Third trimester (weeks 29-42)	3 (8)		—	
	Postpartum	11 (29)		—	
	Infertility	5 (13)		—	

^a^Not applicable.

### Descriptive Statistics

Table 3 shows descriptive statistics for all behavioral and clinical outcomes at baseline, week 2, and week 4. Baseline behavioral data represented 1-3 days of monitoring from screening to baseline assessment, whereas week 2 and week 4 data represented approximately 2-week collection periods. For the step count, distance traveled, and calorie expenditure, baseline (week 0) data were available for 36 (95%), week 2 data for 34 (90%), and week 4 data for 35 (92%) participants. The mean daily step count increased from 2366.8 (SD 2293.0) steps at baseline to 6278.7 (SD 5026.8) steps (*P*<.001) at week 4. The mean distance traveled daily increased from 1564.6 (SD 1528.4) m at baseline to 4105.9 (SD 3117.6) m (*P*<.001) at week 4. The mean daily calorie expenditure increased from 1130.6 kcal (SD 759.9) kcal at baseline to 1834.9 (SD 458.1) kcal (*P*<.001) at week 4. The mean resting heart rate decreased from 85.9 (SD 9.0) bpm at baseline to 80.4 (SD 7.0) bpm (*P*<.01) at week 4. Heart rate data were not available for all participants; the device measures the resting heart rate during sleep periods, but some participants removed the device during sleep or because it had a loose fit. Furthermore, for 36 (95%) participants, mean MADRS scores decreased from 21.3 (SD 5.2) at baseline to 12.3 (SD 8.5) at week 4, and mean BDI scores decreased from 24.8 (SD 11.4) at baseline to 16.1 (SD 11.2) at week 4.

**Table 3:** Descriptive statistics of clinical and behavioral outcomes at each visit.

Variables			Week 0 (baseline)							Week 2 (midintervention)							Week 4 (postintervention)				
			Mean (SD)		Median (IQR)		Participants, n (%)		Mean (SD)			Median (IQR)		Participants, n (%)		Mean (SD)			Median (IQR)		Participants, n (%)
**Clinical outcomes**																					
	MADRS^a^	21.3 (5.2)		20.5 (7.8)		36 (95)		14.6 (7.0)			14.0 (8.0)		36 (95)		12.3 (8.5)			12.0 (10.0)		36 (95)	
	BDI^b^	24.8 (11.4)		21.0 (12.8)		36 (95)		18.3 (11.9)			17.0 (16.0)		36 (95)		16.1 (11.2)			13.5 (19.3)		36 (95)	
**Behavioral outcomes**																					
	Step count	2366.8 (2293.0)		1977.0 (3158.8)		36 (95)		6099.3 (4611.9)			5475.0 (5506.0)		34 (90)		6278.7 (5026.8)			5218.0 (5247.0)		35 (92)	
	Distance traveled	1564.6 (1528.4)		1294.0 (2079.8)		36 (95)		4021.3 (3048.6)			3556.0 (3663.3)		34 (90)		4105.9 (3117.6)			3387.0 (3459.0)		35 (92)	
	Calorie expenditure	1130.6 (759.9)		1568.0 (1335.8)		36 (95)		1771.5 (520.3)			1821.0 (252.8)		34 (90)		1834.9 (458.1)			1790 (317.5)		35 (92)	
	Resting heart rate	85.9 (9.0)		84.7 (8.2)		27 (71)		82.6 (8.2)			80.8 (9.9)		28 (74)		80.4 (7.0)			82.5 (7.1)		27 (71)	

^a^MADRS: Montgomery-Åsberg Depression Rating Scale.

^b^BDI: Beck Depression Inventory.

### Clinical Outcomes: Model-Based Analyses

Table 4 presents results from the LMM analyses of clinical scales. Results indicated significant reductions in clinical scales over time. MADRS scores decreased at week 2 (β=−6.51, *P*<.001, 95% CI −7.59 to −5.44) and at week 4 (β=−8.80, *P*<.001, 95% CI −9.88 to −7.72) compared with baseline. Similarly, BDI scores decreased at week 2 (β=−6.60, *P*<.001, 95% CI −8.01 to −5.19) and at week 4 (β=−8.64, *P*<.001, 95% CI −10.05 to −7.22) compared with baseline.

**Table 4:** Clinical outcomes analyzed with LMMs^a^.

Variable and comparisons		Regression coefficient β (SE)	95% CI	Effect size (Cohen d)	*P* value
**MADRS^b^**					
	Week 2 vs week 0	–6.51 (0.55)	–7.59 to –5.44	1.33	<.001
	Week 4 vs week 0	–8.80 (0.55)	–9.88 to –7.72	1.8	<.001
**BDI^c^**					
	Week 2 vs week 0	–6.60 (0.721)	–8.01 to –5.19	0.95	<.001
	Week 4 vs week 0	–8.64 (0.721)	–10.05 to –7.22	1.2	<.001

^a^LMM: linear mixed model.

^b^MADRS: Montgomery-Åsberg Depression Rating Scale.

^c^BDI: Beck Depression Inventory.

### Behavioral Outcomes: Model-Based and Nonparametric Analyses

**Figure 1:**
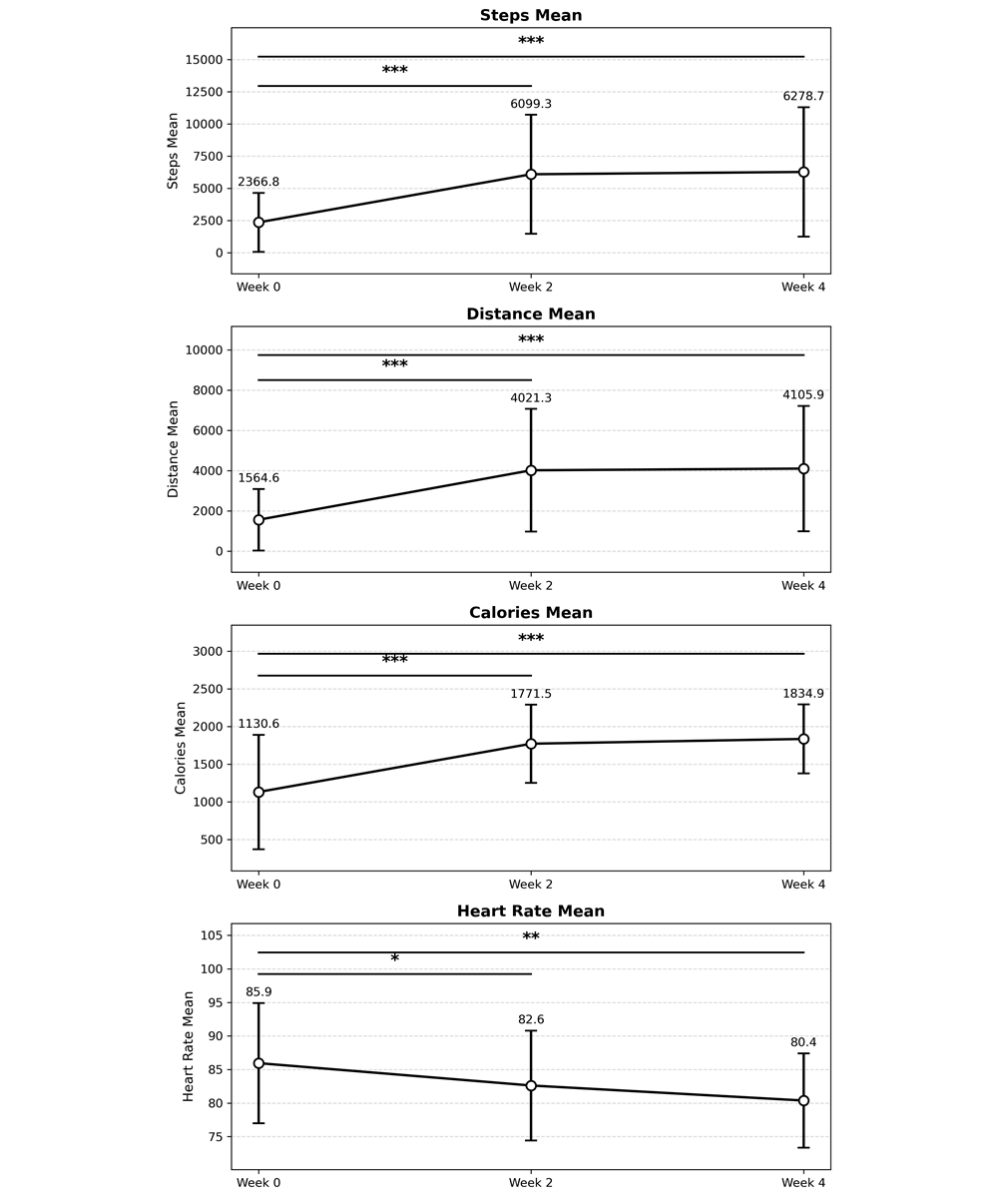
Changes in behavioral outcomes over 4 weeks of the tDCS intervention. Data are presented as the mean (SD). Statistical significance between time points was assessed using Wilcoxon signed-rank tests with Bonferroni correction. *P<.05, **P<.01, ***P<.001. Error bars represent the SD. n=34-36 (89%-95%) for steps, distance, and calories; n=27-28 (71%-74%) for heart rate due to device removal or loose fit during sleep. tDCS: transcranial direct current stimulation.

Figure 1 illustrates the model-estimated temporal trajectories of behavioral outcomes across the three assessment points. LMM analyses confirmed significant time effects for all behavioral indicators. Compared to baseline, the step count increased at week 2 (β=3735.98, *P*<.001, 95% CI 2089.65-5382.32) and week 4 (β=3938.59, *P*<.001, 95% CI 2306.69-5570.48), the distance traveled increased at week 2 (β=2458.66, *P*<.001, 95% CI 1412.46-3504.86) and week 4 (β=2559.28, *P*<.001, 95% CI 1522.32-3596.24), and calorie expenditure increased at week 2 (β=643.18, *P*<.001, 95% CI 380.08-906.28) and week 4 (β=705.99, *P*<.001, 95% CI 445.01-966.97). In contrast, heart rate decreased at week 2 (β=−3.39, *P*=.013, 95% CI −6.06 to −0.71) and week 4 (β=−5.61, *P*<.001, 95% CI −8.33 to −2.90).

Complementary nonparametric analysis results are summarized in Table 5. Friedman tests showed significant effects of time on the step count (*χ*²_2_=24.76, *P*<.001), distance traveled (*χ*²_2_=24.64, *P*<.001), calorie expenditure (*χ*²_2_=22.53, *P*<.001), and heart rate (*χ*²_2_=7.90, *P*=.019). Post hoc Wilcoxon signed-rank tests showed significant changes from baseline to week 2 and from baseline to week 4 for the step count, distance traveled, and calorie expenditure (all *P*<.001), but we observed no significant changes between weeks 2 and 4 (step count: *P*=.75; distance traveled: *P*=.77; calorie expenditure: *P*=.97). Heart rate decreased significantly from baseline to week 2 (*P*=.02) and from baseline to week 4 (*P*=.004), with no significant change between weeks 2 and 4 (*P*=.35). Effect sizes ranged from 0.74 to 0.86 for the step count, from 0.74 to 0.90 for the distance traveled, and from 0.76 to 0.86 for calorie expenditure. For heart rate, effect sizes were −0.57 at week 2 and −0.73 at week 4 compared with baseline.

**Table 5:** Behavioral outcomes across time points (nonparametric tests and effect sizes).^a^

Variable and comparisons		Friedman *P*	Wilcoxon *P*		Cohen d
**Step count**					
	Week 0 vs week 2	<.001	<.001	0.74	
	Week 0 vs week 4	<.001	<.001	0.86	
	Week 2 vs week 4	.75	.75	0.06	
**Distance traveled**					
	Week 0 vs week 2	<.001	<.001	0.74	
	Week 0 vs week 4	<.001	<.001	0.9	
	Week 2 vs week 4	.77	.77	0.05	
**Calorie expenditure**					
	Week 0 vs week 2	<.001	<.001	0.76	
	Week 0 vs week 4	<.001	<.001	0.86	
	Week 2 vs week 4	.97	.97	0.11	
**Heart rate**					
	Week 0 vs week 2	.02	.02	–0.57	
	Week 0 vs week 4	.02	.004	–0.73	
	Week 2 vs week 4	.35	.35	–0.29	

^a^Wilcoxon signed-rank tests were conducted for pairwise comparisons following a significant Friedman test. All *P* values were adjusted for multiple comparisons using Bonferroni correction.

### Correlations Between Clinical and Behavioral Changes

Associations between changes in clinical scales and behavioral indicators are displayed in Table 6. No significant correlations were observed between changes in MADRS scores and behavioral measures across week 0 vs week 2, week 0 vs week 4, or week 2 vs week 4. However, significant correlations were identified for BDI scores, indicating that reductions in BDI scores are associated with increases in behavioral activity. Between baseline and week 2, decreases in BDI scores were correlated with increases in the step count (r=0.401, *P*=.02) and distance traveled (r=0.387, *P*=.02). Between baseline and week 4, similar correlations were observed for the step count (r=0.351, *P*=.04) and distance traveled (r=0.338, *P*=.05). Between weeks 2 and 4, changes in BDI scores were correlated with changes in heart rate (r=0.525, *P*=.01), whereas associations with the step count (r=0.295, *P*=.09) and distance traveled (r=0.293, *P*=.09) were not statistically significant.

**Table 6:** Correlations between changes in clinical scales (MADRS^a^, BDI^b^) and behavioral indicators.

Scale and comparison		Step count		Distance traveled			Calorie expenditure			Heart rate	
		Correlation coefficient r	*P* value	Correlation coefficient r	*P* value	Correlation coefficient r		*P* value	Correlation coefficient r		*P* value
**MADRS^a^**											
	Week 2 vs week 0	0.057	.75	0.034	.85	–0.126		.48	0.007		.98
	Week 4 vs week 0	0.261	.13	0.249	.15	0.114		.52	–0.232		.30
	Week 4 vs week 2	0.159	.37	0.125	.48	0.095		.60	0.228		.28
**BDI^b^**											
	Week 2 vs week 0	0.401	.02	0.387	.02	0.149		.40	0.182		.41
	Week 4 vs week 0	0.351	.04	0.338	.05	0.169		.33	–0.235		.29
	Week 4 vs week 2	0.295	.09	0.293	.09	0.278		.11	0.525		.01

^a^MADRS: Montgomery-Åsberg Depression Rating Scale.

^b^BDI: Beck Depression Inventory.

### Adherence to Intervention

Overall adherence to the tDCS protocol was favorable across sites. Among the 38 participants, the mean completion rate was 68.9% (SD 28.3%) when considering full sessions only and 73.3% (SD 26.3%) when partial sessions were also included.

When adherence was examined based on the number of completed full sessions (out of 28 scheduled sessions), 22 (58%) participants completed at least 20 (71%) sessions, 10 (26%) participants completed 10-19 (36%-68%) sessions, and 6 (16%) participants completed fewer than 10 (<36%) sessions. Using a threshold of ≥70% adherence, 22 (58%) participants met this criterion based on full sessions only, and 25 (66%) participants met the same threshold when partial sessions were included.

Site-level adherence results and detailed distributions are summarized in Multimedia Appendix 3.

### Safety and Tolerability

The tDCS intervention was generally well tolerated. A total of 10 (26%) participants experienced AEs during the 4-week intervention, most of which were mild to moderate in severity.

The most frequent AEs were skin and subcutaneous tissue disorders, including pruritus (n=1, 3%), skin irritation (n=5, 13%), and rash (n=1, 3%). Nervous system disorders were reported in 3 (8%) participants, all including mild headaches. Importantly, one serious adverse event (SAE) occurred, corresponding to a single case of skin irritation that met the criteria for seriousness (investigator-judged as requiring temporary medical attention but without hospitalization or lasting sequelae).

All other events were nonserious and transient, and no participants discontinued treatment due to AEs. Details of the AE profile are summarized in Table 7.

**Table 7:** Summary of reported AEs^a^ during the 4-week tDCS^b^ intervention.

Symptom category and reported symptom			Participants with AEs, n (%)	Participants, n (%)		
				Non-SAE^c^		SAE
**Skin and subcutaneous tissue disorders**						
	Pruritus	1 (3)		1 (3)	0	
	Skin irritation	5 (13)		4 (11)	1 (3)	
	Rash	1 (3)		1 (3)	0	
**Nervous system disorders**						
	Headache	3 (8)		3 (8)	0	
Total participants			10 (26)	9 (24)		1 (3)

^a^AE: adverse event.

^b^tDCS: transcranial direct current stimulation.

^c^SAE: serious adverse event.

## Discussion

### Principal Findings

To the best of our knowledge, this is the first study to combine self-administrated tDCS and continuous wearable data in perinatal women. This pilot study investigated associations between 4 weeks of daily tDCS and changes in depressive symptoms and wearable-derived behavioral outcomes in perinatal women. We observed concurrent reductions in depressive symptoms, increases in physical activity markers, and decreases in heart rate during the intervention period. Notably, reductions in self-reported BDI scores were correlated with changes in behavioral indicators, while clinician-rated MADRS scores were not, suggesting differential patterns in how these assessment modalities relate to real-world behavioral changes. These findings demonstrate the feasibility of integrating wearable monitoring with tDCS protocols across a heterogeneous perinatal population and provide preliminary evidence supporting further investigation in randomized controlled trials.

Both MADRS and BDI scores improved significantly across the study period, indicating meaningful symptom reduction. These results are consistent with prior evidence for tDCS in perinatal women. Previous randomized controlled trials have reported reductions in depressive symptoms among pregnant women without major maternal or neonatal complications [14]. Open-label studies during pregnancy have similarly demonstrated substantial improvements in depressive symptoms, with no obstetric or neonatal adverse outcomes [15]. Furthermore, systematic reviews of noninvasive brain stimulation during pregnancy have highlighted the promise and tolerability of tDCS in this population [17]. Together, these findings strengthen the evidence base that tDCS may be considered a safe and potentially beneficial intervention for perinatal depression, and our results extend prior work by demonstrating consistent improvements across both clinician-rated (MADRS) and self-reported (BDI) scales in a single study cohort.

Wearable-derived outcomes showed significant increases in step counts, distances traveled, and calorie expenditure, accompanied by decreases in heart rate. These improvements were evident by week 2 and were sustained through week 4. Such objective data provide an ecologically valid complement to traditional symptom ratings by reflecting day-to-day functioning in real-world contexts [22]. In neurological populations, repeated sessions of tDCS have been associated with cumulative improvements in motor and functional outcomes, suggesting that stimulation can enhance behavioral performance, in addition to potentially reducing symptoms [34]. In this study, the improvements in wearable-based outcomes paralleled the symptom reductions, underscoring the added value of integrating digital phenotyping into clinical trials. However, the findings reflect exploratory associations, and further work is needed to determine how wearable-derived measures can be systematically evaluated and applied within clinical trial frameworks. Digital phenotyping research has further shown that wearable-based indicators are sensitive to concurrent changes in self-reported mood and stress, reinforcing their utility as objective proxies of functional improvement [24,25].

It should be noted that baseline activity levels were particularly low, with a mean of approximately 2400 steps/day. Although this may partly reflect the shorter baseline collection period (1-3 days vs 2 weeks at follow-up), it also aligns with prior evidence that perinatal depression commonly presents with marked reductions in physical activity and behavioral activation. The substantial increases observed by week 2 and sustained through week 4 suggest that tDCS may be associated with behavioral activation in this population, although the uncontrolled design precludes definitive causal inference [35,36].

Exploratory analyses revealed that reductions in BDI scores, but not MADRS scores, were significantly associated with increases in step counts and distances traveled, as well as with decreases in heart rate. This pattern suggests that self-reported symptoms may capture subjective experiences that align more directly with daily activity patterns, whereas clinician-administered assessments may reflect broader, standardized clinical constructs with different relationships to real-world behavioral changes. This interpretation is supported by recent digital health research, which has shown that self-reported measures collected via mobile platforms are more strongly associated with wearable-derived indicators of activity than clinician-rated assessments [24]. Our findings add to this growing body of evidence, suggesting that patient-reported outcomes, when combined with objective wearable monitoring, may provide a more ecologically valid picture of functional change. From a clinical perspective, this highlights the importance of considering multiple outcome modalities to capture both symptom severity and functional change in perinatal populations. However, given the exploratory nature of these analyses and the number of comparisons conducted, these findings should be interpreted cautiously and require replication in larger controlled samples.

The intervention was well tolerated, and only 1 participant experienced an SAE (skin irritation requiring brief medical attention), with no treatment discontinuations or long-term effects. This observation is consistent with previous trials, in which tDCS during pregnancy or postpartum was associated with good tolerability and an absence of major maternal or neonatal complications [14-16]. A Cochrane review of tDCS across various populations similarly concluded that the rate of AEs and treatment discontinuation was comparable to sham stimulation, providing broader evidence of the safety of this intervention [12]. Recent trials of home-based or remotely supervised tDCS for depression have also demonstrated that this modality is both feasible and acceptable, further supporting the scalability of tDCS interventions in routine practice [37]. Our results contribute to this literature by showing that wearable monitoring can be effectively combined with stimulation in perinatal women, adding an additional layer of feasibility for future trials and demonstrating the potential for integrated digital health approaches in this population. Home-based, self-administered tDCS may represent a scalable intervention approach that could help address behavioral health provider shortages in perinatal care settings, pending validation in controlled trials.

### Limitations

Several limitations should be acknowledged. First, and most importantly, the absence of a sham control group is a critical limitation of this pilot study. Without a control condition, observed changes cannot be attributed specifically to tDCS, as they may reflect placebo effects, natural symptom fluctuation, regression to the mean, or the effects of increased monitoring and supportive contact. Perinatal depression is known to improve spontaneously in some cases, particularly when women receive regular attention and support [38]. Therefore, no causal claims can be made from the findings, and results should be interpreted as preliminary associations that require validation in randomized sham-controlled trials to establish whether tDCS causally improves symptoms and functioning.

Second, several measurement challenges affected the wearable monitoring data. Baseline behavioral data were collected over a shorter period (1-3 days) compared to follow-up assessments (approximately 2 weeks), which may have contributed to artificially low baseline activity values and limits interpretation of the magnitude of behavioral changes. Additionally, heart rate data had substantial missingness due to device removal during sleep or loose device fit. Because no formal statistical test was performed to confirm the missing-at-random assumption, heart rate findings should be interpreted as exploratory. Although we considered this to represent missing at random with respect to treatment response, the representativeness of these data remains uncertain, and the Fitbit device measures the resting heart rate primarily during sleep periods, which represents a specific physiological state. Future studies should use consistent measurement periods across all time points and optimize device-wearing protocols to minimize data loss.

Third, the relatively small sample size limited statistical power and precluded meaningful subgroup analyses. The sample was heterogeneous, including women experiencing infertility, women across different pregnancy trimesters, and postpartum women, which limits interpretability regarding perinatal stage–specific effects. These groups may differ in their baseline characteristics, symptom profiles, and responses to intervention. However, the pilot design could not adequately address such differences. Future larger trials should consider stratification by perinatal status to examine potential differential effects.

Fourth, the exploratory correlation analyses involved multiple comparisons. Although we applied Bonferroni corrections to pairwise Wilcoxon tests, several significant correlations between BDI changes and behavioral indicators had *P* values near the significance threshold (eg, .04, .05). These findings should be interpreted cautiously and require replication in independent samples before firm conclusions can be drawn about differential sensitivity of assessment modalities.

Fifth, the study was limited to a 4-week intervention period, and therefore, the durability of the observed symptom reductions and behavioral improvements remains unknown. Longer-term follow-up is needed to determine whether benefits are maintained after treatment discontinuation. Additionally, wearable-derived measures, although ecologically valid, may be influenced by contextual factors, such as caregiving responsibilities, occupational demands, or lifestyle variations, which were not systematically controlled in this study.

Finally, effect sizes reported for within-subject pre-post comparisons should be interpreted as descriptive rather than definitive estimates of treatment effects. In the absence of a control group, such effect sizes may be inflated and do not necessarily reflect the true magnitude of tDCS-specific effects. These values are reported primarily to facilitate comparison with future controlled studies.

### Conclusion

In summary, this pilot study provides preliminary evidence that 4 weeks of daily tDCS in perinatal women is associated with significant reductions in depressive symptoms, improvements in daily activity, and decreases in heart rate. Improvements emerge early and can be sustained through the intervention period. Self-reported outcomes (BDI) show stronger associations with wearable-based behavioral indicators compared to clinician-rated outcomes (MADRS), suggesting that patient-reported measures may be particularly sensitive to ecologically valid changes in functioning. The intervention is safe, feasible, and well tolerated, consistent with previous evidence from perinatal and broader clinical populations.

However, the open-label design without a sham control group is a fundamental limitation that precludes causal inference. Rather than prioritizing a single wearable-derived measure, future trials may benefit from evaluating multiple complementary indicators of activity and physiology, with stepwise validation focusing on reproducibility, sensitivity to change, and clinical relevance. The observed changes may reflect tDCS effects but could also result from placebo responses, natural symptom fluctuation, or increased monitoring. Therefore, these findings should be considered hypothesis-generating rather than confirmatory.

These results support the integration of wearable-based monitoring into tDCS trials as a strategy to capture multidimensional outcomes and emphasize the importance of combining clinician-rated, self-reported, and objective measures. Larger randomized sham-controlled trials with consistent measurement periods across time points, adequate statistical power for subgroup analyses, extended follow-up to assess the durability of effects, and stratification by perinatal status (eg, pregnancy trimester, postpartum period) are essential to validate these preliminary associations and establish whether tDCS causally improves symptoms and functioning in perinatal depression. Such trials should continue to incorporate multiple outcome modalities to comprehensively evaluate treatment effects and inform clinical implementation.

## Appendix

Multimedia Appendix 1 Definition of wearable-derived behavioral features.

Multimedia Appendix 2 Sensitivity analyses: age-adjusted LMMs. LMM: linear mixed model.

Multimedia Appendix 3 Site-specific adherence to the tDCS intervention. tDCS: transcranial direct current stimulation.

## Abbreviations

AE: adverse event
API: application programming interface
BDI: Beck Depression Inventory
DLPFC: dorsolateral prefrontal cortex
HAMD: Hamilton Depression Rating Scale
IRB: Institutional Review Board
LMM: linear mixed model
MADRS: Montgomery-Åsberg Depression Rating Scale
MDD: major depressive disorder
SAE: serious adverse event
tDCS: transcranial direct current stimulation

## Data Availability

The datasets generated and/or analyzed during this study are not publicly available because of restrictions containing information that could compromise the privacy of study participants but are available from the corresponding author upon reasonable request.
