# Supplementary material for: Clinical and Behavioral Outcomes During 4 Weeks of Home-Based Self-Administered Transcranial Direct Current Stimulation in Perinatal Women With Depressive Symptoms: Open-Label Exploratory Pilot Study
Source: JMIR Form Res. 2026 Mar 12;10:e56454. doi: 10.2196/56454 (PMC13022536; doi:10.2196/56454)
Supplement: Multimedia Appendix 1 [file formative_v10i1e56454_app1.docx]

**Multimedia Appendix 1. Definition of wearable-derived behavioral features**

| **Feature** | **Source** | **Unit** | **Fitbit data source** | **Description** |
| --- | --- | --- | --- | --- |
| Steps | Wearable activity logs | steps/day | Fitbit API endpoint | \|  \| \| --- \|   Total number of steps recorded while the device is worn, derived from minute-level activity data |
| Distance | Wearable activity logs | meters/day | Activity intraday | \|  \| \| --- \|   Distance estimated by the device based on step counts and user-specific stride length parameters |
| Calories | Wearable activity logs | kcal/day | Activity intraday | \|  \| \| --- \|   Distance estimated by the device based on step counts and user-specific stride length parameters |
| Heart rate | Wearable PPG | beats/min | \|  \| \| --- \|  \| activities-heart-intraday \| \| --- \| | Total energy expenditure, including physical activity and resting metabolic processes, as defined by the manufacturer |

Note. Data were collected using Fitbit Inspire 2 devices.
