# Supplementary material for: Clinical and Behavioral Outcomes During 4 Weeks of Home-Based Self-Administered Transcranial Direct Current Stimulation in Perinatal Women With Depressive Symptoms: Open-Label Exploratory Pilot Study
Source: JMIR Form Res. 2026 Mar 12;10:e56454. doi: 10.2196/56454 (PMC13022536; doi:10.2196/56454)
Supplement: Multimedia Appendix 2 [file formative_v10i1e56454_app2.docx]

**Multimedia Appendix 2. Sensitivity analyses: Age-adjusted linear mixed models**

| **Outcome** | **n** | **Week2 β** | **Week2  95% CI** | | **p** | **Week4 β** | **Week4  95% CI** | | **p** | **Age β** | **Age 95% CI** | | **p** |
| --- | --- | --- | --- | --- | --- | --- | --- | --- | --- | --- | --- | --- | --- |
| steps | 36 | 3684.531 | 2025.359 | 5343.702 | <0.001 | 3872.326 | 2226.216 | 5518.436 | <0.001 | 177.259 | -61.049 | 415.567 | 0.145 |
| distance | 36 | 2426.711 | 1372.636 | 3480.787 | <0.001 | 2518.253 | 1472.564 | 3563.941 | <0.001 | 112.163 | -42.066 | 266.392 | 0.154 |
| calories | 36 | 638.265 | 373.837 | 902.694 | <0.001 | 699.89 | 437.416 | 962.365 | <0.001 | 13.793 | -17.995 | 45.582 | 0.395 |
| heart rate | 33 | -3.362 | -6.04 | -0.684 | 0.014 | -5.589 | -8.303 | -2.874 | <0.001 | -0.152 | -0.697 | 0.393 | 0.585 |
| MADRSscore | 36 | -6.667 | -8.82 | -4.513 | <0.001 | -9 | -11.154 | -6.846 | <0.001 | 0.237 | -0.194 | 0.669 | 0.28 |
| KBDIscore | 36 | -6.528 | -9.277 | -3.779 | <0.001 | -8.667 | -11.415 | -5.918 | <0.001 | -0.265 | -1.035 | 0.505 | 0.5 |

Note. Age_S1_c indicates age centered at the sample mean. Models included a random intercept for participant; time was modeled as a categorical fixed effect (Week0 reference).
