# Supplementary material for: Clinical and Behavioral Outcomes During 4 Weeks of Home-Based Self-Administered Transcranial Direct Current Stimulation in Perinatal Women With Depressive Symptoms: Open-Label Exploratory Pilot Study
Source: JMIR Form Res. 2026 Mar 12;10:e56454. doi: 10.2196/56454 (PMC13022536; doi:10.2196/56454)

**Multimedia Appendix 3. Site-specific adherence to tDCS intervention**

**Table S1. Distribution of completed tDCS sessions (Full sessions only)**

| **Completed sessions**  **(out of 28)** | **Seoul National University Hospital** | **Yongin Severance Hospital** | **Bundang CHA Medical Center** | **Ilsan CHA Hospital** | **Total** |
| --- | --- | --- | --- | --- | --- |
| **n** | 4 | 7 | 8 | 19 | 38 |
| **≥20 sessions** | 3 (75.0%) | 5 (71.4%) | 5 (62.5%) | 9 (47.4%) | 22 (57.9%) |
| **10–19 sessions** | 1 (25.0%) | 1 (14.3%) | 1 (12.5%) | 7 (36.8%) | 10 (26.3%) |
| **<10 sessions** | 0 (0.0%) | 1 (14.3%) | 2 (25.0%) | 3 (15.8%) | 6 (15.8%) |

**Note.** Full sessions were defined as sessions in which the full 30-minute stimulation was completed.

**Table S2. Distribution of completed tDCS sessions (Full or partial sessions)**

| **Completed sessions**  **(out of 28)** | **Seoul National University Hospital** | **Yongin Severance Hospital** | **Bundang CHA Medical Center** | **Ilsan CHA Hospital** | **Total** |
| --- | --- | --- | --- | --- | --- |
| **n** | 4 | 7 | 8 | 19 | 38 |
| **≥20 sessions** | 3 (75.0%) | 6 (85.7%) | 5 (62.5%) | 11 (57.9%) | 25 (65.8%) |
| **10–19 sessions** | 1 (25.0%) | 0 (0.0%) | 1 (12.5%) | 6 (31.6%) | 8 (21.1%) |
| **<10 sessions** | 0 (0.0%) | 1 (14.3%) | 2 (25.0%) | 2 (10.5%) | 5 (13.2%) |

**Note.** Partial sessions were defined as sessions in which ≥70% of the scheduled stimulation duration was completed.

**Figure S1. Overall Distribution of Completed tDCS sessions**


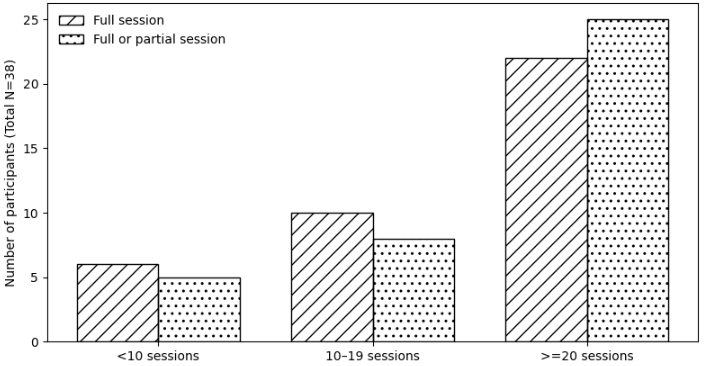

Supplement: Multimedia Appendix 3 [file formative_v10i1e56454_app3.docx]
